# Pharmacogenomics for Primary Care: An Overview

**DOI:** 10.3390/genes11111337

**Published:** 2020-11-12

**Authors:** Victoria Rollinson, Richard Turner, Munir Pirmohamed

**Affiliations:** Wolfson Centre for Personalised Medicine, Institute of Systems, Molecular and Integrative Biology, University of Liverpool, Liverpool L69 3GL, UK; rt34@liverpool.ac.uk (R.T.); munirp@liverpool.ac.uk (M.P.)

**Keywords:** pharmacogenomics, primary care, adverse drug reaction, implementation, antidepressants, warfarin, statins, clopidogrel, drug hypersensitivity

## Abstract

Most of the prescribing and dispensing of medicines happens in primary care. Pharmacogenomics (PGx) is the study and clinical application of the role of genetic variation on drug response. Mounting evidence suggests PGx can improve the safety and/or efficacy of several medications commonly prescribed in primary care. However, implementation of PGx has generally been limited to a relatively few academic hospital centres, with little adoption in primary care. Despite this, many primary healthcare providers are optimistic about the role of PGx in their future practice. The increasing prevalence of direct-to-consumer genetic testing and primary care PGx studies herald the plausible gradual introduction of PGx into primary care and highlight the changes needed for optimal translation. In this article, the potential utility of PGx in primary care will be explored and on-going barriers to implementation discussed. The evidence base of several drug-gene pairs relevant to primary care will be outlined with a focus on antidepressants, codeine and tramadol, statins, clopidogrel, warfarin, metoprolol and allopurinol. This review is intended to provide both a general introduction to PGx with a more in-depth overview of elements relevant to primary care.

## 1. Introduction

It is increasingly recognised that individuals respond differently to medications. In some cases, these differences in response can be clinically significant leading to failure of therapy or adverse drug reactions (ADRs). The aetiology of this inter-individual variability is complex with multiple contributors including individual characteristics (e.g., age, sex, body mass index), clinical factors (e.g., renal or hepatic impairment; co-medications), environmental exposures (e.g., smoking) and genetics.

Pharmacogenomics (PGx) is the study of the influence of genetic variation on drug response [1] with the aim of increasing the efficacy and safety of current and future treatments. Specifically, it aims to facilitate a move away from the standard empirical trial and error prescribing approach that currently exists and transition towards a more stratified and precise prescribing paradigm.

**Figure 1:**
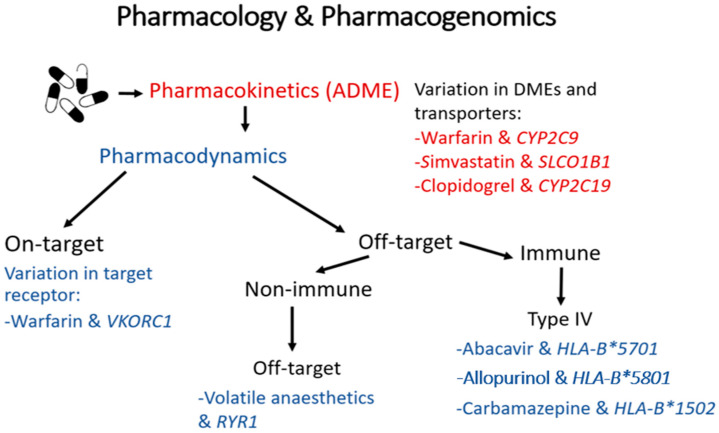
A summary of the mechanisms by which genomic variation can influence the pharmacokinetics and/or pharmacodynamics of a drug, with examples of clinically relevant drug-gene pairs. Genetic changes in drug-metabolising enzymes and transporters can alter a drug’s pharmacokinetics, influencing its tissue exposure and so affecting its downstream pharmacodynamics. These changes can lead to underexposure and potentially reduced effectiveness, or increase exposure predisposing often to type A (augmented) adverse drug reactions (ADRs), which are ADRs mediated through excessive action on the drug’s therapeutic target and so are predictable (e.g., bleeding after excessive warfarinisation). Secondly, genetic polymorphisms in a drug’s therapeutic target can directly modulate drug response, leading to reduced effectiveness or type A ADRs. Lastly, many small molecule drugs bind with varying affinities to unintended off-target molecules, both within and separate to the immune system. Genetic changes in these off-target sites can predispose to less predictable and less common type B (bizarre) ADRs. Of particular note, many rare but serious type B ADRs, such as drug-induced skin injury and drug-induced liver injury, are type IV T-cell mediated delayed type hypersensitivity reactions, and have been very strongly associated with specific human leukocyte antigen (HLA) alleles. It should also be noted that changes in a drug’s pharmacokinetics leading to increased exposure can, on occasion, predispose to off-target (type B) ADRs, as is the case, for example, with simvastatin myotoxicity.

It is estimated that there are between 19,000 to 21,000 protein-coding genes present in the human genome [2]. Within these genes, multiple types of genetic variations can occur including single nucleotide polymorphisms (SNPs), indels (small insertion/deletions) and larger structural rearrangements; of these, SNPs are the most common. Pharmacokinetics (PK) describes “what the body does to a drug” and pharmacodynamics (PD) “what a drug does to the body”. Genomic variation in genes involved in a drug’s absorption, distribution, metabolism and elimination (e.g., drug-metabolising enzymes or transporters) can alter a drug’s PK profile, influencing systemic exposure and resulting in altered drug response (i.e., influencing its downstream PD). Alternatively, genomic variation in genes that modulate a drug’s PD (e.g., its therapeutic on-target and off-target sites) can directly influence drug response. Importantly, in both cases, the altered drug response can attenuate a drug’s efficacy or worsen tolerability/safety (Figure 1).

The clinical and financial consequences of adverse drug reactions (ADRs) are high, accounting for an estimated 6.5% of hospital admissions [3]. Interestingly, for several of the drugs often implicated in causing hospitalisation, PGx guideline recommendations are now available, such as for warfarin, antiplatelet agents and opioid analgesics [4]. In addition to serious ADRs leading to hospitalisation, it is also known that poor drug tolerability (for example due to mild adverse events) is associated with lower compliance [5], which increases the chances of reduced efficacy and increased medicines wastage.

It is estimated that over 98% of individuals carry at least one pharmacogenetic variant [6]. Importantly, the majority of prescribing and dispensing of medicines happens in primary care, and recent studies in both the US and UK suggest that over 60% of patients within the primary care setting are prescribed a medication with a PGx recommendation [7,8]

The Dutch Pharmacogenomics Working Group (DPWG) and Clinical Pharmacogenetics Implementation Consortium (CPIC) are the two most widely recognised expert groups involved in the development of PGx clinical guidelines. Therapeutic recommendations from CPIC and DPWG, as well as guidelines written by other groups such as the Canadian Pharmacogenomics Network for Drug Safety (CPNDS), are curated and housed by the Pharmacogenomics Knowledge Base (https://www.pharmgkb.org/), and can be accessed online for free by healthcare professionals and other individuals with an interest. To date, PGx associations with actionable PGx recommendations for clinical practice have been developed for just over 80 drugs [9]. Almost two-thirds of these actionable drug-gene associations involve drug metabolizing enzyme genes, with ~80% of these being genes encoding cytochrome P450 (CYP) enzymes. A small number of actionable drug-gene associations involve transporter genes, and just under a third involve genes that influence drug PD (~5% on-target, ~26% off-target, with almost a third of the latter involving human leukocyte antigen (*HLA*) genes). The US Food & Drug Administration (FDA) has also evaluated drug-gene associations, deeming 47 drugs to have sufficient evidence for PGx therapeutic recommendations and another 16 drugs to have PGx associations that potentially impact clinical safety or response [10]; there is notably overlap between the drug-gene associations considered actionable by the guideline committees and the FDA.

A list of drugs (though not exhaustive) commonly prescribed in primary care with PGx guidelines currently available is outlined in Table 1.

Despite the growing availability of guidelines, adoption of PGx in primary care has been slow. Nevertheless, a nationwide study from the Netherlands inferred that 1 out of every 19 new prescriptions in primary care could have undergone an adjustment had PGx data been available [11]. Within the UK and in many other countries, PGx testing largely remains within the remit of specialist secondary and tertiary care settings (e.g., with abacavir-HLA B*57:01 testing). Table 2 provides a summary, albeit non-exhaustive, of recent and large interventional studies that have assessed PGx and are relevant to primary care.

A recent analysis identified the following pharmacogenes to be most commonly linked to primary care prescriptions in England: *CYP2D6*, *CYP2C19* and *SLCO1B1* (solute carrier organic anion transporter family member 1B1), followed by *CYP2C9*, *VKORC1* (vitamin K epoxide reductase complex 1), *CYP4F2* and *HLA-B* (8).Therefore, the subsequent sections in this review provide an overview of the role PGx can play in primary care in the prescribing and monitoring of specific medications related to these genes. Drug-specific literature searches were conducted between June and October 2020.

## 2. Antidepressants

Prescribed for a variety of indications, the number of prescriptions for antidepressants in the UK primary care sector is increasing annually [21]. Whilst they are effective therapies, these drugs have variable success rates [22], with up to 50% of patients nonresponsive to treatment. Some of this heterogeneity in response may be accounted for by PGx variation.

In the UK, selective serotonin re-uptake inhibitors (SSRIs) account for over 50% of primary care antidepressant prescriptions [23]; tricyclic antidepressants (TCAs), serotonin-norepinephrine reuptake inhibitors (SNRIs) and atypical antidepressants (e.g., mirtazapine) are also utilised.

There are 57 putatively functional CYP genes within the human genome, of which around 12 are involved in the biotransformation of 70–80% of all therapeutics used in clinical practice [24]. Most antidepressants are, in some part, metabolized via the CYP enzyme system, with CYP2D6 and CYP2C19 [25] widely regarded as the most influential enzymes for antidepressant biotransformation.

*CYP2D6* is highly polymorphic with over 100 allelic variants recorded [26]. Due to the number of possible diplotypes within a population the translation of genotype to phenotype for *CYP2D6* is often performed using an “activity score” wherein alleles are given a numeric value based on their functionality and the sum of the values present in an individual is subsequently categorised into one of the following “metaboliser phenotypes”: ultra-rapid metabolizer (UM), extensive (normal) metabolizer (EM), intermediate metabolizer (IM), and poor metabolizer (PM) [27].

Allelic frequencies in *CYP2D6* vary substantially across populations. The most common non-functioning variant, CYP2D6*4 (rs3892097, 1846G > A), which is a splicing defect, is found at the highest frequency within Caucasian populations [28]; related to this observation Caucasian populations have a higher frequency of PMs (around 5–10%) compared to Asian and African-American populations. CYP2D6 is also subject to copy number variation (CNV) that can lead to deletion of a CYP2D6 allele (*5) or CYP2D6 duplication or multiplication; whilst the former decreases CYP2D6 function, an increased number of functional CYP2D6 alleles results in the UM phenotype (*xN). UMs are less frequent in Caucasian populations at approximately 2%, but the frequency of UMs has been noted to be much higher in other specific populations, such as those with North African ancestry (c.25%) [29].

Studies have demonstrated that the presence of the CYP2D6*4 allele in PMs correlated to increased side effect frequency with some antidepressants including venlafaxine [30] and lower dose requirements of SSRIs and TCAs (e.g., amitriptyline, nortriptyline; see below) [31]. Additionally, studies have indicated that *CYP2D6* UMs taking paroxetine have low plasma concentrations in comparison to EMs [32] and this may be a risk factor for therapeutic failure. In the case of both fluvoxamine and paroxetine, the CPIC guideline highlights the possibility of ADRs potentiated by higher plasma concentrations in *CYP2D6* PMs [33]. Some evidence exists to correlate increased ADRs with mirtazapine in *CYP2D6* IM and PM patients [34,35]. However, at this time, no clinically actionable PGx recommendation exists for mirtazapine.

*CYP2C19* is similarly highly polymorphic: 34 conventional star alleles are currently recognised [36] and over 2000 *CYP2C19* variants have been identified, although the majority are intronic [37]. The most prevalent reduction-of-function (ROF) allele is *2 (rs4244285, c.681G > A), a splice defect, followed by *3 (rs4986893, c.636G > A) which results in a premature stop codon. CYP2C19*2 is most common in individuals of Asian ancestry with minor allele frequencies (MAFs) of 31–36% [38], whilst occurring in ~16% of African and European individuals. CYP2C19*3 occurs in ~6% of East Asian individuals, but is rare in African and European populations. Conversely, CYP2C19*17 (rs12248560), which is a common variant in the promotor region of the gene, is associated with increased transcription and enzymatic function [39].

Similar to *CYP2D6*, individuals can be categorised according to genotype into anticipated CYP2C19 EMs (*1/*1 wild-type), IMs (one ROF allele), PMs (two ROF alleles), and UMs (*17/*17). Unlike conventional *CYP2D6* phenotype categorisation, a *CYP2C19* rapid metaboliser (RM, *1/*17) phenotype category is also recognised with functionality between EMs and UMs.

Sertraline, citalopram and escitalopram are all extensively metabolised by CYP2C19 and, in PMs, elevated plasma concentrations may lead to potential ADRs [40,41]. Presently, however, a formal drug label warning only exists for escitalopram, which recommends a reduced starting dose for *CYP2C19* PMs due to the risk of QT interval prolongation [42].

Amitriptyline undergoes CYP2C19-mediated demethylation to its active metabolite, nortriptyline, which is also available on prescription. Whilst both are classed as TCAs, amitriptyline blocks serotonin and noradrenaline reuptake equally, whereas nortriptyline inhibits noradrenaline uptake more potently [43]. As a result, *CYP2C19* PMs are expected to show reduced conversion to nortriptyline, increased systemic exposure to amitriptyline and an increased risk of amitriptyline-related side effects [44]. Although TCAs are now more commonly used at lower doses for the treatment of neuropathic pain, rather than for major depressive disorder, the CPIC guidelines for amitriptyline still recommend consideration of alternative therapy in *CYP2C19* UMs due to an increased risk of treatment failure irrespective of indication [45]. Additionally, amitriptyline and nortriptyline also undergo *CYP2D6* metabolism to a less active metabolite. Whereas *CYP2C19* influences the ratio of metabolites, *CYP2D6* plays a greater role in drug clearance. This may lead to raised plasma concentrations of amitriptyline and nortriptyline in CYP2D6 PMs or in patients co-administered a CYP2D6 inhibitor [46]. CPIC guidelines therefore recommend a lower starting dose when used at dosing ranges for depression [47].

A recent meta-analysis of five randomised clinical trials (RCTs) reported that patients receiving PGx guided dosing were 1.71 (95% CI: 1.17–2.48, *p* = 0.005) times more likely to achieve symptom remission for major depressive disorder (MDD) than those on standard care [48]. Details of some of the most recent RCTs are in Table 2.

While there is still work to be done on the utilisation of PGx in antidepressant prescribing, there are multiple potential benefits to having PGx information at hand when selecting treatments. An analysis of UK healthcare data from 2016 identified that the age categories with highest antidepressant use were ages 55–64 and 75–84 [49]. Additionally, the same data set demonstrated an increasing trend for polypharmacy with increasing age, particularly in the over 75-years-old age bracket. PGx testing has the potential to circumvent some of the risks associated with polypharmacy by guiding the prescribing of suitable medications [50].

In addition to patient benefits, analyses have shown that utilising PGx information in mental health prescribing may offer significant financial savings via a reduction in the number of failed treatments and subsequent medicines wastage [51].

## 3. Opioid Analgesics

Codeine and tramadol are commonly prescribed weak opioid analgesics indicated for mild to moderate (non-neuropathic) pain; they are positioned on the second rung of the World Health Organisation’s three step pain ladder. Codeine is an inactive prodrug; 0–15% undergoes O-demethylation catalysed by CYP2D6 to form active morphine, which has an affinity for the µ-opioid receptor 200-fold more than that of parent codeine [52]. Similarly, tramadol undergoes CYP2D6-mediated O-demethylation to O-desmethyltramadol [53], which has an affinity for the µ-opioid receptor that is also significantly higher than that of tramadol [54]. Whereas the majority of codeine’s analgesic effect stems from morphine agonism on opioid receptors, for tramadol, the analgesia conferred by O-desmethyltramadol agonism of the µ-opioid receptor is complemented by inhibition of serotonin and noradrenaline reuptake by parent tramadol.

In *CYP2D6* EM individuals, at least 80% of codeine metabolism results in inactive metabolites, with around 5–10% undergoing biotransformation into morphine [55]. This percentage increases in UMs [56] whilst, in PMs, there may be very little if any conversion (52). PK and PD studies have demonstrated that the increased codeine biotransformation in *CYP2D6* UMs can result in opiate toxicity even with low doses of codeine [57]. Conversely, *CYP2D6* PMs are likely to obtain little to no therapeutic benefit [58]. In addition, due to the heterogeneity in *CYP2D6* allele functionality, some patients within the broad EM category may also experience an increased number of adverse events compared with others. These “ultra-rapid EMs” have alleles leading to a greater activity score, but which still fall within the EM activity score reference range, and thus ideally require lower doses to circumvent possible ADRs [59].

The PGx of codeine received international attention following reports of infant opiate toxicity from higher levels of morphine in the breast milk of UM mothers. Reviews of this phenomenon resulted in amended FDA and European Medicines Agency (EMA) guidance to avoid the use of codeine when breastfeeding [60].

Similarly, regulatory changes followed case reports of children with obstructive sleep apnoea receiving codeine post-tonsillectomy and/or adenoidectomy leading to codeine toxicity and fatalities, with these children having evidence of being *CYP2D6* UMs [61,62]. This led to blanket regulation from authorities such as the UK Medicines and Healthcare products Regulatory Agency (MHRA) [63] restricting the use of codeine in children under 12 irrespective of *CYP2D6* metaboliser status. This same MHRA guidance extends to cover all patients (i.e., 12 years and older) with a documented *CYP2D6* UM status, although in practice this is seldom known. Both CPIC and the DPWG have developed PGx guidelines for codeine that advocate avoiding codeine in UM and PM patients. However, there are nuanced differences between these recommendations. Most pertinently, in UMs, CPIC recommends simply avoiding codeine, whereas the DPWG guidance suggests no additional action is required in UM patients that receive lower doses of codeine and have no additional risk factors (e.g., concomitant CYP3A4-inhibiting drugs) that would predispose to exaggerated biotransformation to morphine.

In addition to the influence of *CYP2D6*, there is emerging evidence for the influence of polymorphisms within other genes including the µ-opioid receptor gene, *OPRM1*, and the phase 2 metabolising enzyme *UGT2B7* [64]. While associations with variation in these genes remain inconclusive, they are important areas for future research.

Within the UK, the use of the weak opioid tramadol has increased over the past two decades [65]. In keeping with codeine, studies have demonstrated a probable reduced efficacy with tramadol in *CYP2D6* PMs patients and, additionally, a lower risk of ADRs [66]. Again, the risk of ADRs including potentially severe reactions appears greater in UMs [67,68]. A systematic review of the CYP2D6*10 C188T polymorphism which included 9 studies and 809 subjects showed a relationship between this polymorphism and pharmacokinetics of tramadol (half-life, AUC and clearance) and analgesic effect, but not with the adverse effects of nausea and vomiting [69].

Although CPIC has yet to develop guidance for tramadol, the DPWG guidelines recommend avoiding tramadol in UMs or, if not possible, using just 40% of the standard dose, to minimise the risk of opioid receptor-mediated ADRs. For PMs, an increased alertness to reduced effectiveness is primarily recommended. This is because it is difficult to predict the influence of decreased CYP2D6 metabolism on the overall analgesic effects experienced by an individual, because the resulting increased proportion of parent tramadol to O-desmethyltramadol may enable the potentiated SNRI properties of parent tramadol to mitigate some of the lost O-desmethyltramadol opioid receptor-mediated analgesic effects.

## 4. Statins

Statins are first line lipid-lowering agents for both primary and secondary prevention of cardiovascular disease, are widely prescribed, and are generally safe and well tolerated. Statins are, however, associated with an increased risk of type 2 diabetes mellitus and muscle toxicity, which is phenotypically heterogeneous ranging from myalgias with normal plasma creatine kinase (CK, ~5% of individuals [70]) to infrequent myopathies to rare rhabdomyolysis, and finally extremely rare immune-mediated necrotizing myopathy (IMNM) [71]. A recent study [72] showed that the risk of myopathy (CK > 10 × the upper limit of normal) with simvastatin was 9 per 10,000 person-years of therapy. Independent risk factors for myopathy included simvastatin dose, ethnicity, sex, age, body mass index, medically treated diabetes, and concomitant use of certain drugs (niacin-laropiprant, verapamil, β-blockers, diltiazem and diuretics), which collectively predicted more than a 30-fold risk difference between the top and bottom thirds of a clinical myopathy score. This risk score was less strongly associated with milder myopathy, and not associated with reports of any muscle symptoms, and so the authors concluded that muscle presentations other than myopathy were unlikely to be related to statins [73].

Statins competitively inhibit 3-hydroxy-3-methylglutaryl-Coenzyme A reductase (HMGCR) in the liver, resulting in decreased circulating low-density lipoprotein cholesterol (LDL-C). Organic anion-transporting polypeptide 1B1 (OATP1B1) is a liver-specific transporter expressed on the basolateral (sinusoidal) side of hepatocytes [74], is encoded by *SLCO1B1* and is involved in hepatic uptake of statins and other drugs (e.g., letermovir [73]). A common ROF *SLCO1B1* missense variant, rs4149056 (c.521T > C, p.V174A, present in SLCO1B1*5, *15 and *17), is associated with a 221% increased systemic exposure to simvastatin acid in 521CC compared to wild-type 521TT individuals. Moreover, the C allele is associated with elevated exposures to all statins, except fluvastatin, albeit to lesser extents [75]. For example, the corresponding increase with atorvastatin is 145%. The MAF of rs4149056 in African, East Asian and European populations is 1%, 12% and 16%, respectively.

Importantly, it was shown in a genome-wide association study (GWAS) that 521TC and 521CC patients taking simvastatin 80mg daily had an odds ratio (OR) for myopathy of 4.5 (95% confidence interval (CI) 2.6–7.7) and 16.9 (95% CI 4.7–61.1) compared to wild-type patients, respectively [76]. In 521TC patients on simvastatin 40mg, the relative risk was 2.6 (95% CI 1.3–5.0). Furthermore, the 521C allele is associated with a two-fold increase in statin intolerance in patients predominantly taking simvastatin, and this intolerance can reduce lipid-lowering efficacy [77]. Thus, the strength of *SLCO1B1*-myotoxicity association increases with 521C allele dose, simvastatin dose and myopathy severity.

Several other studies [78] including a recent large-scale GWAS [79] have replicated the simvastatin-521T > C association. The consistent association with simvastatin mirrors the PK observations that 521T > C has the largest impact on simvastatin exposure, reflects the high prevalence of simvastatin use in clinical practice leading to reliance on simvastatin myotoxicity cases in research studies, and the in vitro observations that simvastatin (lactone) is particularly myotoxic [80]. Elevated simvastatin systemic exposure presumably increases skeletal muscle exposure, predisposing to myotoxicity by mechanisms that are incompletely understood but likely to include mitochondrial dysfunction, calcium signalling disruption and reduced prenylation.

The evidence for an association between SLCO1B1 521T > C and other statins is less clear. Some studies have suggestively associated 521C with atorvastatin myotoxicity [81,82,83], but others found no association [84,85,86]. Interestingly, a recent study in patients on high dose atorvastatin for secondary prevention found 521C was associated with both muscular symptoms and atorvastatin intolerance, suggesting that 521T > C may be more relevant in patients on high dose (e.g., 80 mg) atorvastatin [87]. For rosuvastatin, 521C was not associated with myalgias in patients of European descent [88], but recently has been associated with myotoxicity (myalgias to rhabdomyolysis) in Chinese ancestry patients [89]. *SLCO1B1* 521T > C has not been associated with pravastatin myotoxicity.

A recent international whole-exome sequencing endeavour of patients with statin (most commonly simvastatin) myopathy identified no novel variants [90]. Nevertheless, a candidate missense variant (rs12975366, p.D247G) within leukocyte immunoglobulin-like receptor subfamily B member 5 (*LILRB5*) was recently associated with statin myotoxicity, including intolerance and myalgia, implicating the immune system in these more mild phenotypes [91]; an intervention study is underway [92]. Moreover, CYP3A4*22 and *CYP3A5* non-expressors have been associated with modestly elevated simvastatin exposure [93], CYP3A7*1C with increased atorvastatin hydroxylation [89], and *ABCG2* rs2231142 (c.421C > A) with elevations in exposure to simvastatin, atorvastatin, fluvastatin and especially rosuvastatin [75,94]. Nevertheless, despite signals, these candidate genes and others (e.g., *ABCB1*) have not been consistently associated with statin myotoxicity across studies. Similarly, *CYP3A4* [95], *CYP3A5* [96] and *CYP2D6* [97] have been investigated as potential biomarkers of statin efficacy, but results have varied and, of note, none were identified in a GWAS meta-analysis of statin lipid-lowering efficacy [98]. Nevertheless, the influence of more complex interactions, such as those involving combined genetic variation in both *SLCO1B1* and *CYP* and/or other transporter genes, on statin pharmacokinetics and clinical effects, warrants further study. In addition, *CYP2C9* ROF variants have been associated with increased adverse events (primarily myotoxicity [99]) and potentially increased lipid-lowering efficacy [100]. Fluvastatin has arguably received less research attention than simvastatin and atorvastatin, yet *CYP2C9* is important in fluvastatin’s metabolism, and so follow up studies are warranted to further investigate these findings.

*SLCO1B1* 521T > C has been associated with the spectrum of myotoxicity, at least for simvastatin, except for IMNM. A subtype of IMNM is anti-HMGCR myopathy [101]; most patients with anti-HMGCR myopathy have a history or statin exposure, develop muscle weakness with highly elevated CK levels that persist despite statin cessation, are identified by the presence of circulating anti-HMGCR autoantibodies that can be directly pathogenic, and normally require treatment with immunosuppressive drugs or intravenous immunoglobulins [102]. Interestingly, HLA-DRB1*11:01 has been significantly associated with anti-HMGCR myopathy [103,104] with estimated OR of ~25–57 dependent on ethnicity. Whilst this HLA association may catalyse research into underlying mechanisms and aid diagnosis, the rarity of anti-HMGCR myopathy suggests this PGx association will not have utility in guiding statin initiation.

Both CPIC and the DPWG have developed clinical guidelines for simvastatin-SLCO1B1 [105,106], and the DPWG have also developed atorvastatin-SLCO1B1 guidance. In patients that carry 521C, they recommend starting an alternate statin or using a lower simvastatin dose. For atorvastatin, the DPWG guideline recommends an alternative statin in 521C carriers that have additional clinical myotoxicity risk factors. Nevertheless, few patients are now started on simvastatin 80 mg following an FDA warning about its increased myopathy risk [107]. Interestingly, a recent randomized trial in 159 patients with previous statin myalgia has demonstrated that providing *SLCO1B1* 521T > C genotype with recommendations increases statin re-initiation in primary care [108]. As muscle symptoms are associated with statin discontinuation and non-adherence [5], which in turn increase the risk of cardiovascular events [109], 521T > C genotyping may help reduce cardiovascular events as well as myotoxicity in clinical practice, although this remains to be substantiated.

## 5. Clopidogrel

Clopidogrel is an antiplatelet drug indicated in acute coronary syndrome (ACS), percutaneous coronary intervention (PCI), stroke, transient ischaemic attack (TIA), peripheral artery disease (PAD) and atrial fibrillation (AF). Clopidogrel is a prodrug: of the ~50% absorbed, ~85% is rapidly hydrolysed to inactive clopidogrel carboxylic acid via carboxylesterase 1 (CES1) and ~15% undergoes a two-step oxidative biotransformation to produce the active thiol metabolite that irreversibly inhibits platelet P2Y12 receptors [110].

CYP2C19 is the only CYP substantially involved in both oxidative steps, contributing 45% and 21% to the first and second steps, respectively [111]. *CYP2C19* ROF alleles are associated with decreased levels of circulating clopidogrel active metabolite [112] and increased ex vivo high on-treatment platelet reactivity (HTPR) [113]. A meta-analysis of 9685 patients, of whom 91% had undergone PCI (55% ACS), demonstrated that *CYP2C19* ROF alleles were associated with an increased risk of major adverse cardiovascular events (MACE) and stent thrombosis, with a gene-dose trend evident [114]. However, a second meta-analysis assessing *CYP2C19* genotype and cardiovascular outcomes in 26, 251 individuals found no overall association with cardiovascular outcomes after exclusion of small studies, although an increased risk of stent thrombosis remained evident [115]. This meta-analysis included a wider spectrum of indications for clopidogrel, including atrial fibrillation. To resolve these discrepant observations, clopidogrel indication-specific PGx has been posited. Thus, these (mostly) observational data have been considered to support an association between *CYP2C19* ROF variants and cardiovascular events in post-PCI patients, because this patient population’s high baseline risk for cardiovascular events makes them more susceptible to suboptimal treatment, but does not support an association in other (mainly cardiac) settings where the overall benefit of clopidogrel is modest anyway [116].

To investigate whether interventions based on *CYP2C19* ROF alleles improve clinical outcomes, two large randomized clinical trials (RCTs) have been undertaken and recently reported (Table 2) [18,19]. The Patient Outcome after primary PCI (POPular) Genetics trial included 2488 patients with an ST-elevation myocardial infarction (STEMI) that underwent primary PCI with stent insertion and compared standard-treatment (ticagrelor or prasugrel) to a genotype-informed antiplatelet strategy that allocated ticagrelor/prasugrel to CYP2C19*2 or *3 carriers and clopidogrel to noncarriers. POPular Genetics reported the genotype strategy was non-inferior to standard-treatment for net adverse clinical events (MACE plus major bleeding, *p* < 0.001 for noninferiority), but reduced (mostly minor) bleeding (hazard ratio (HR) 0.78, 95% CI 0.61–0.98, *p* = 0.04). The Tailored Antiplatelet Initiation to Lessen Outcomes Due to Decreased Clopidogrel Response after PCI (TAILOR-PCI) RCT [18] recruited 5302 patients undergoing PCI with stent insertion (for ACS or stable coronary artery disease) and compared standard-treatment clopidogrel to an equivalent CYP2C19-informed antiplatelet strategy to that used in POPular Genetics. The primary result of TAILOR-PCI was a reduction in first MACE in the genotype strategy arm compared to the standard (clopidogrel) arm, although it narrowly missed statistical significance (HR 0.66, 95% CI 0.43–1.02, *p* = 0.06). A pre-specified sensitivity analysis showed a significant reduction in all cumulative MACE events (HR 0.60, 95% CI 0.41–0.89, *p* = 0.01) with genotyping. There was no difference in bleeding risk between arms. Of note, the power to detect a difference in TAILOR-PCI was lower than originally planned because the event rate was lower than anticipated, in keeping with use of newer generation drug-eluting stents [18].

CYP2C 19*17 has been associated with decreased HTPR, and inconsistently with increased bleeding risk and reduced MACE [117]. Given the inconsistent findings, less observational research has focused on *17 compared to *CYP2C19* ROF alleles, and that *17 was not included in the POPular Genetics or TAILOR-PCI primary analyses, the clinical relevance of *17 remains undetermined.

There has been an increased focus recently on the impact of *CYP2C19* ROF alleles in patients following a stroke. A recent meta-analysis of studies of patients prescribed clopidogrel for ischaemic stroke/TIA found *CYP2C19* ROF carriers had an increased risk of recurrent stroke and MACE [118]. *CYP2C19* ROF alleles have also been associated with increased in-stent restenosis in patients with PAD [119]. On the other hand, a genetic analysis of the large EUCLID trial (*n* = 13,885) that recruited patients with symptomatic PAD reported no difference in cardiovascular outcomes between patient subgroups with different *CYP2C19* genotypes [120]. However, EUCLID excluded *CYP2C19* PM patients from entry into the trial, limiting interpretation of these findings.

Beyond *CYP2C19*, there is growing evidence for a role of *CES1* in clopidogrel PGx. *CES1* catalyses clopidogrel to its inactive metabolite; the variant allele of rs71647871 (p.G143E, MAF ~1%) in *CES1* has been associated with increased circulating clopidogrel active metabolite, reduced ex vivo platelet reactivity, and a nonsignificant trend towards decreased MACE [121]. Moreover, the variant allele of CES1 rs2307240 (p.S75N, MAF ~5%) was recently associated with reduced subsequent MACE in clopidogrel-treated ACS patients [106].

Both CPIC [121] and DPWG (106) have produced clinical guidelines for clopidogrel-CYP2C19: CPIC focuses on ACS patients undergoing PCI, and DPWG on PCI for any indication, as well as stroke and TIA. The main recommendation is alternative antiplatelet therapy in those with *CYP2C19* ROF alleles, although the DPWG guidance also permits doubling the dose of clopidogrel in *CYP2C19* IMs. A multi-site real world implementation of *CYP2C19* genotyping to guide antiplatelet stratification after PCI (*n* = 1815) has been reported, and importantly demonstrated clinical benefit [122]. On balance, taking all evidence together, we contend that clinical implementation of clopidogrel-CYP2C19 genotyping is justifiable and beneficial.

## 6. Warfarin

The oral vitamin K antagonist, warfarin, is indicated to prevent and treat venous thromboembolism (VTE) and to prevent thromboembolism in atrial fibrillation (AF) and following mechanical heart valve transplantation. Warfarin is a racemate that competitively inhibits VKORC1 within the vitamin K cycle, leading to hypofunctional clotting factors II, VII, IX and X. The extent of anticoagulation is measured by the international normalised ratio (INR) and, for most indications, the target therapeutic INR is 2.0–3.0 [123]. Warfarin stable dose (WSD) requirements vary ~30-fold between patients [124], and its narrow therapeutic index underlies warfarin being the third most common drug to lead to hospitalisation due to ADRs(3). A 10% increase in time outside the therapeutic INR range (TTR) is associated with increased thromboembolic events and mortality [125], and supratherapeutic INRs increase the risk of major bleeding [126].

Overall, ~60% of the observed variation in warfarin stable dose requirements can be explained, and several clinical factors including age, smoking and interacting drugs contribute [127]. However, genetic factors explain the majority of this observed variation. Specifically, rs9923231 (-1639G > A) in *VKORC1* accounts for 6–25% of observed variation, variation in *CYP2C9* another ~15%, and CYP4F2*3 (rs2108622, p.V433M) 1–7% [128,129,130].

-1639G > A lies within *VKORC1′s* promoter region; -1639A alters a transcription factor binding site reducing *VKORC1* transcription [131], increasing sensitivity to warfarin and reducing WSD requirements [132]. The MAF of -1639A in African, East Asian, South Asian and European populations is ~5%, ~90%, ~15% and ~40%, demonstrating allele reversal in East Asians. The variation in -1639A MAF perhaps underlies why -1639G > A explains only 6% of WSD variation in African-Americans but ~20–25% in Asian and Caucasian populations [133].

CYP2C9 metabolises the more potent S-warfarin enantiomer and *CYP2C9* ROF variants reduce WSD requirements. CYP2C9*2 (rs1799853, p.R144C) and *3 (rs1057910, p.I359L) are common ROF alleles in European populations with MAFs of ~12% and ~7%, respectively, and CYP2C9*3 is common in Asian populations (MAF 3–11%). However, CYP2C9*2 is infrequent in Asians (0–3%) and both are rare or infrequent in African populations (0–3.6% for *2, and 0.3–2% for *3) [134]. Interestingly, distinct warfarin-associated variants have been described in African ancestry patients (primarily Africa-Americans). CYP2C9*5, *6, *8 and *11 are ROF alleles with a collective frequency of ~20% in African ancestry individuals [135], but are rare in other ethnicities. All of these variants, except CYP2C9*8, have been confirmed by meta-analysis to reduce warfarin dose requirements in black African patients [136]. The non-coding variant, rs12777823, within the *CYP2C* cluster upstream of *CYP2C18*, has also been associated with reduced WSD requirements, independent of *CYP2C9* [137].

CYP4F2 mediates removal of active (reduced) vitamin K from the vitamin K cycle. CYP4F2*3 is associated with lower hepatic CYP4F2 [138] and increased WSD requirements in Asian [139] and European ancestry patients, but not in those of African ancestry [140,141].

Beyond *VKORC1/CYP2C9/CYP4F2*, many other candidate genes have been investigated for their role in variable warfarin dose requirements, although findings have been mostly contradictory, and not supported by GWAS. Interestingly however, rs7856096 in the folate homeostasis gene, folylpolyglutamate synthase (*FPGS*), was identified in African-Americans by exome sequencing and replicated [142]. It decreases *FPGS* transcription and is associated with reduced WSD requirements, although the causative mechanism remains unknown.

Several RCTs have investigated the utility of genetic-guided warfarin dosing algorithms (Table 2). The largest trial to date, the Genetic Informatics Trial (GIFT), recruited patients initiating warfarin for elective hip or knee surgery [17]. GIFT compared 808 patients that received genotype-guided dosing (considering *VKORC1* -1639G > A, CYP2C9*2, CYP2C9*3, CYP4F2*3) to 789 that received clinically-guided dosing. The primary clinical composite endpoint of major bleeding, INR ≥ 4, VTE or death occurred in 10.8% of patients in the genotyped arm versus 14.7% in the clinically-guided arm (*p* = 0.02). The high-risk subgroup of patients (those with a difference of at least 1.0mg/day between clinically guided and genotype-predicted warfarin doses), which plausibly had a higher number of warfarin sensitive alleles, particularly benefitted from genotyping with a larger (7%) increase in TTR. The findings in GIFT were consistent with the EU-PACT RCT, but not with COAG (Table 2) [15,16,131]

Both CPIC [143] and DPWG have developed warfarin PGx guidelines for commencing warfarin that recommend validated genetic algorithms (e.g., the EU-PACT loading algorithm [15] or International Warfarin Pharmacogenetics Consortium (IWPC) algorithm [144]) or percent dose alterations. Importantly, point-of-care genotype-guided warfarin dosing has been shown to significantly improve TTR following implementation in real world anticoagulation clinics [145], and appears cost-effective [16].

The majority of patients investigated have been of European ancestry.As described above, ethnicity influences the prevalence of warfarin risk alleles, particularly in those of Sub-Saharan African origin. Thus, ethnicity-specific algorithms have been developed, although prospective testing of these algorithms is currently limited [146,147]. Moreover, self-reported ethnicity may not represent genetic ancestry well, especially for admixed individuals [148]. Nonetheless, it has been recently shown that an individual’s genetic ancestry can be reasonably determined from clinically focused PGx SNP panels (122 and 243 SNPs) compared with genome-wide genotyping [149]. This approach could stratify individual’s by genetic ancestry to facilitate implementation of ethnicity-specific warfarin algorithms.

Although warfarin remains commonly prescribed, it is clear that the use of direct oral anticoagulants (DOACs) is increasing [150]. DOACs are at least as safe and effective as warfarin in the prevention of stroke in non-valvular AF [151] and monitoring is not required. However, their current high price restricts access to them in many healthcare systems, and concerns over long term adherence [152], as well as patient choice, mean they are not suitable for all patients. Interestingly, bleeding risk appears equivalent between DOACs and warfarin in patients who have no *VKORC1*/*CYP2C9* warfarin risk alleles [153] or their anticoagulation centre-based TTR is ≥ 66% [154], and thus anticoagulant stratification based on *VKORC1*/*CYP2C9* genotypes has been posited [155]. Moreover, no DOAC is indicated following mechanical heart valve surgery or in those with a creatinine clearance < 15mL/min; thus, further research into the utility of warfarin PGx in these specific settings is warranted.

## 7. Metoprolol

Metoprolol is a racemate cardioselective β1-adrenoreceptor blocking agent used for indications including hypertension, heart failure, angina pectoris, arrhythmias and migraine prophylaxis. Around 70% of an oral dose undergoes metabolism by CYP2D6 to inactive metabolites [156]. Plasma levels of metoprolol have been found to be significantly higher (around 4–5 ×) in *CYP2D6* PMs compared with EMs [157].

Metoprolol decreases cardiac output via negative chronotropic, as well as inotropic effects, thus reducing heart rate [158]. Retrospective and prospective studies have demonstrated significantly greater reductions in heart rate in PMs vs. non-PMs [159,160] and an increased risk of bradycardia [161]. Although, there is observed heterogeneity in the incidence of bradycardia between studies, a recent meta-analysis identified that overall the prevalence of bradycardia is statistically significantly higher in PMs [162]. Nevertheless, the clinical relevance of this finding is presently unclear, given that many of the instances of bradycardia have been asymptomatic (e.g., detected on an electrocardiogram performed as part of a study protocol) with comparatively little reported around symptomatic bradyarrhythmias.

The DPWG guideline for metoprolol in CYP2D6 PM and IM patients suggest a dose reduction (to 25% and 50% of the standard dose, respectively) where a gradual reduction in heart rate is required (for example, in chronic heart failure) or symptomatic bradycardia occurs. As there may be increased conversion to inactive metabolites in UM patients blunting the efficacy of metoprolol, dose increases beyond the usual maximum dose or selection of an alternative β-blocker (indication dependent) may be required [106]. While other β-blockers including carvedilol, nebivolol and propranolol are also metabolized by CYP2D6, the extent of their CYP2D6-dependent metabolism is less than for metoprolol [163]. Moreover, atenolol is not metabolised, and bisoprolol undergoes balanced elimination with half excreted via the kidneys and the other half undergoing metabolism by CYP3A4 and CYP2D6; CYP2D6 does not appear to be an important predictor of bisoprolol exposure or function [164,165].

## 8. Allopurinol

The xanthine oxidase inhibitor, allopurinol, is commonly prescribed in primary care for the prophylaxis of gout; it is also indicated in other hyperuricaemic conditions including tumour lysis syndrome. Although allopurinol is generally well tolerated, severe cutaneous adverse reactions (SCARs) represent a rare (0.1–0.4% of patients) but serious allopurinol ADR. The SCARs, Stevens–Johnson syndrome (SJS), toxic epidermal necrolysis (TEN), and drug reaction with eosinophilia and systemic symptoms (DRESS), have all been reported following allopurinol exposure [166].

SCARs have overlapping clinical features including eosinophilia, hepatic/renal dysfunction and rash or skin detachment. SJS and TEN are considered to represent different severities along a continuum of the same disease process; SJS is diagnosed when ≤ 10% body surface area is affected, TEN when > 30% is affected, and an overlap syndrome occurs with 10–30% body surface area involvement [167]. Allopurinol is the most common cause of SCARs in Europe [168]. Furthermore, SCARs have high morbidity and mortality rates [167,169] and the mortality specifically from allopurinol-associated SCARs has been reported to approach 25% [168,170].

Studies have shown strong associations between allopurinol-related SCARs and carrying HLA-B*58:01 [171,172]. In an interventional cohort study in Taiwan, genotyping HLA-B*58:01 and giving alternate treatment to HLA-B*58:01 positive patients, whilst HLA-B*58:01 negative patients received allopurinol, resulted in no SCAR cases, compared to an estimated seven cases based on the historical average (*p* = 0.0026) [20].

The allopurinol SCAR-*HLA-B* association is considered to be a type IV hypersensitivity reaction and is T-cell mediated [173]; typically, such reactions occur up to 3 months after drug initiation (most cases occurring within 8–9 weeks [174] and are delayed in onset (as opposed to anaphylactic reactions with other drugs which are immediate) [175]. Other identified clinical factors that increase the risk of allopurinol SCAR include older age, female sex, chronic kidney disease and higher starting doses, which all plausibly increase systemic exposure [176]. Following oral administration, allopurinol is almost completely metabolised into its active metabolite, oxypurinol, which has an elimination half-life of 15 h, compared to 1–2 h for allopurinol [177]. Regarding immunopathogenesis, both allopurinol and oxypurinol have been shown to directly bind HLA-B*58:01 and Arg97 of HLA-B*58:01 is a plausible binding site for oxypurinol via hydrogen bond formation [178]. Oxypurinol-sensitive T-cell lines can have mixed populations of CD4+ and CD8+ T-cells. Moreover, in vitro T-cell activation assays after culturing peripheral blood mononuclear cells from patients with previous allopurinol-associated SCARs demonstrated that oxypurinol increased granulysin in a concentration- and time-dependent manner, but allopurinol and febuxostat did not, indicative of oxypurinol-induced T-cell activation [179]. Sequencing the T-cell receptors demonstrated that clonotype-specific T-cells that secrete granulysin in response to oxypurinol likely participate in the pathogenesis [179]. Overall, these observations are consistent with the p-i hypothesis (direct pharmacological interaction between a drug/metabolite and HLA molecules) whereby allopurinol-associated SCARs are the result of MHC Class I-driven activation of oxypurinol-specific T-cell clones through an antigen-processing independent route.

The frequency of HLA-B*58:01 varies with ethnicity, but occurs at a higher prevalence in East Asian populations [180]. *HLA-B* expression is co-dominant and individuals need only carry one copy of HLA-B*58:01 to be at increased risk of SCARs. Accordingly, genotyping results are usually reported as HLA-B*58:01 positive, where one of more copies of the allele are detected, or negative where the allele is not detected. This association is now mentioned within the European drug label and CPIC guidelines for allopurinol advise against its use in known HLA-B*58:01 carriers [170,181]

More generally, associations between specific *HLA* alleles and serious ADRs due to at least 24 different drugs have now been reported [182], highlighting the importance of the *HLA* locus to immune-mediated type IV hypersensitivity reactions. Like, allopurinol, several of these associations are very strong (e.g., odds ratios in the tens-thousands) and related to drug-induced skin injury, such as between carbamazepine-HLA-B*15:02 and SJS-TEN, and carbamazepine-HLA-A*31:01 that is associated with both specific SCARs (DRESS and SJS-TEN) and more mild but common (up to 10% of patients [183]) maculopapular exanthema [184]. *HLA* alleles have also been associated with drug-induced liver injury, such as between flucloxacillin and HLA-B*57:01 [185]. Of note, HLA-B*57:01 is also associated with abacavir hypersensitivity syndrome [186]. As previously mentioned, HLA-DRB1*11:01 has been significantly associated with statin-related anti-HMGCR myopathy [103]. These associations provide mechanistic insight and can aid in preventing or diagnosing a serious ADR.

## 9. Transitioning to the Future

**Figure 2:**
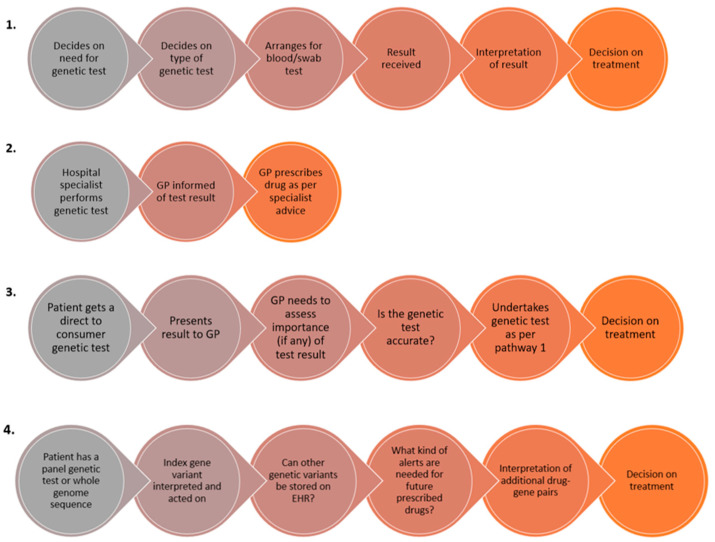
Four hypothetical pathways through which GPs may encounter pharmacogenetic tests within their primary care practice. Pathway 1 is where the GP initiates the test themselves; pathway 2 is where the genetic test is carried out in secondary care and the GP is informed of the test result; and pathway 3 is where the patient presents to the GP having undertaken a test privately or via a direct to consumer genetic testing laboratory. Pathways 1–3 represent situations where a single gene/variant test is undertaken for one gene-drug pair. Pathway 4 represents the situation where the patient has undergone a panel test or exome/whole genome sequencing, where there is the added complexity of storing the rest of the genetic data and having the ability to retrieve it, and use it appropriately in a pre-emptive fashion when a patient is prescribed a new drug(s).

The current literature demonstrates that many primary healthcare providers including physicians and pharmacists are hopeful about the role of PGx in enhancing the care of their patients [187,188] but often highlight that there are still multiple barriers impeding translation into daily practice [189]. Potential pathways of how GPs may encounter PGx tests are shown in Figure 2. Recent studies have identified that barriers to PGx implementation specifically within primary care include a perceived lack of evidence for clinical utility, unclear cost effectiveness and reimbursement strategies, how to educate the primary care workforce regarding PGx, unclear roles and responsibilities particularly between general practitioners and pharmacists, the need for informatics to support PGx-informed clinical prescribing decisions, and concerns over the principles of data sharing as well as other ethical, legal and social implications (ELSI) surrounding PGx [190,191].

Efforts to overcome these barriers and facilitate PGx translation are ongoing. Within Europe, the Ubiquitous Pharmacogenomics Consortium (U-PGx) is undertaking a large, international implementation project (PREemptive Pharmacogenomic testing for prevention of Adverse drug REactions (PREPARE)) within both primary and secondary care settings [192]. This study aims to recruit around 7000 patients to determine the clinical utility and cost effectiveness of testing a panel of 44 PGx variants in 12 genes relevant to 42 drugs for which a DPWG PGx guideline has been developed; the primary focus of PREPARE is to investigate whether PGx implementation reduces ADRs within the first 12 weeks of starting one of these 42 drugs, compared to standard care. Similar initiatives adopting panel-based testing are ongoing around the world, including the Pharmacogenomic Resource for Enhanced Decisions in Care and Treatment program (PREDICT) in the US, which has pre-emptively tested over 10,000 patients to date [6]. Moreover, in the UK, the NHS Genomic Medicine Service (GMS) outlined its plans to have genomic medicine fully integrated into routine practice by 2025 [193].

Despite the cost of genetic tests decreasing, cost-effectiveness remains a concern for PGx testing. While some drug-gene pairs, such as abacavir-HLA-B*57:01 and allopurinol-HLA-B*58:01, have demonstrated cost-effectiveness compared with no genetic testing, others such as factor V Leiden testing prior to oral contraception, have shown mixed and inconclusive results [194]. Panel testing is the most common form of PGx testing currently being utilised in large scale studies such as PREPARE, and is likely more cost effective than single gene testing [195]. Although panel testing does not offer the same in-depth information for a gene compared to whole genome sequencing, its upfront costs are less, test turnaround times are shorter, and interpretation of gene regions is less complicated, which arguably makes it a better suited technology for larger scale implementation in the near to medium term.

Patient focus groups have shown a preference for a familiar healthcare provider to be involved with delivering PGx services [196]. In addition, the uptake of direct-to-consumer testing is increasing and so there is a need to upskill the primary care workforce at large to improve its genetic literacy [197]. It is clear that wider implementation of PGx into the primary care sector will be soon upon us, and it is essential that all areas are able to access the resources to become more comfortable with PGx and overcome the aforementioned barriers.

One critical area for implementation is the development of appropriate clinical decision support systems (CDSS) that facilitate use of PGx information at the point of prescribing and, ideally, integrate this information with other factors routinely considered in the prescribing decision-making process, such as co-medications and comorbidities. In the UK at least, the widespread and early adoption of electronic medical patient records in primary care, which are provided by only a handful of service providers (e.g., EMIS and SystemOne), provide a pre-existing technological infrastructure to build PGx CDSS into. In contrast, the varying uptake of electronic records in secondary care and the greater heterogeneity in providers poses additional barriers to implementing PGx in hospital settings.

As the implementation infrastructure for PGx is gradually erected, it will be important to transition to healthcare learning systems where clinical and research activities are more clearly linked. Thus, ideally, novel clinically relevant and validated discoveries can then be implemented in a shorter time frame than currently, and the effects in the real-world of new interventions observed, measured and iteratively fed back to the research arm to inform future enquiry. A key component of an effective healthcare learning system will be merging presently disparate data sources together and putting systems in place to efficiently collect future clinical data as it is generated to enable big data analyses, and there is little reason why PGx cannot be at the forefront of such endeavours. For example, combining primary care records with array genetic data at scale within UK Biobank has enabled recent PGx analysis investigating interactions between 200 drugs and nine genes in 200,000 subjects, leading to confirmation of several established drug-gene pairs, as well as providing genetic evidence of more novel associations such as between citalopram and reduced incidence of herpes zoster in *CYP2C19* IMs [198].

It should be noted that, to date, the majority of PGx associations involve common variants, including the drug-gene variant associations highlighted in this review. Rare variants (i.e., those with a minor allele frequency < 1%) are often missed in small clinical studies or GWAS analyses, but some may have large effects on outcomes [199]. Case studies of rare and serious ADRs have highlighted the clinical impact of rare variation in pharmacogenes. For example, a recent case report detailed development of reversible encephalopathy and coma after a paediatric patient received a single dose of ivermectin, attributable to compound heterozygosity from two nonsense mutations in *ABCB1*, which encodes P-glycoprotein [200]. Furthermore, the increasing use of next generation sequencing (NGS) technologies in national and other large genomic medicine projects heralds a new era for investigating rare variation at scale. It is currently estimated that, overall, 10–40% of genetic functional variation in pharmacogenes is attributable to rare ght to account for 9% and 39% of the functional variation in *SLCO1B1* and *ABCC1*, respectively variation, although it varies considerably between genes [201]. For example, rare variation is thou Machine learning and other advanced methodologies will likely be needed to parse this rich sequence data, particularly when combined with other data types (e.g., clinical and other omics datasets), to aid functional interpretation and variant discovery, refine genotype-to-phenotype predictions, stratify patient groups, and predict drug response [202,203].

## 10. Conclusions

The majority of prescribing happens in primary care. Owing to interindividual variation, some patients experience reduced effectiveness and others ADRs. Extensive research into PGx has identified and replicated multiple drug-gene pairs, mainly associated with ADRs, with several of the associated drugs commonly prescribed in primary care. The ongoing development of PGx guidelines by CPIC, DPWG and others offers a standardised approach to translating drug-gene associations into actionable prescribing recommendations based on the collective evidence available. Nevertheless, several barriers still exist to wider adoption of PGx into primary care, although these are gradually being addressed. Moving forward, a transition towards healthcare learning systems that harness big data and advanced analytical techniques with implementation of clinically relevant findings is anticipated to advance patient care. One area for focus is the surge in multimorbidity and related polypharmacy that multiple primary care and wider healthcare systems are facing. Although further research is required, it is envisaged that PGx will play a meaningful role in optimising medicines and managing polypharmacy [50]. Overall, it is hoped that implementation of PGx in primary care will be a major activity over the next five years, with ongoing PGx discoveries further boosting the case for implementation.

## Figures and Tables

**Table 1:** Commonly used drugs in primary care with available pharmacogenomics guidelines.

Class	Drug	Gene	Actionable Result	Guideline Availability		Therapeutic Recommendations ^1^
				DPWG	CPIC	
Lipid lowering agents	Atorvastatin	*SLCO1B1*	rs4145096 (521T > C) carriers	√	-	AD
	Simvastatin	*SLCO1B1*		√	√	LD, AD, M
Antidepressants	Citalopram	*CYP2C19*	PM, UM	√	√	LD (PM), AD (PM, UM)
	Sertraline	*CYP2C19*	PM, UM	√	√	LD (PM), AD (PM, UM)
	Amitriptyline	*CYP2C19* *CYP2D6*	PM, RM, UM IM, PM, UM	- √	√ √	LD (PM), AD (PM, RM, UM) LD (IM, PM), AD (PM, UM)
Analgesics	Codeine	*CYP2D6*	IM, PM, UM	√	√	AD (UM, PM), M (IM)
	Tramadol	*CYP2D6*	IM, PM, UM	√	-	AD (IM, PM, UM), ID (IM, PM), LD (UM)
Anti-platelet	Clopidogrel	*CYP2C19*	IM, PM	√	√	AD
Anticoagulant	Warfarin	*VKORC1* *CYP2C9* *CYP2C* region	*VKORC1 c.*-1639G > A *2, *3, *5, *6, *8, *11 rs12777823	√ √	√ √ √	LD ^2^ LD ^2^ LD
Anticonvulsant	Carbamazepine	*HLA-B* *HLA-A*	HLA-B*15:02 detected HLA-A*31:01 detected	- -	√ √	AD, M AD, M
Antibiotic	Flucloxacillin	*HLA-B*	HLA-B*57:01 detected	√	-	AD, M
Contraception	Oestrogen-containing contraceptives	*F5*	rs6025 (p.R534Q) carriers	√	-	AD
Xanthine oxidase inhibitor	Allopurinol	*HLA-B*	HLA-B*58:01 detected	-	√	AD

^1^ = Where a drug-gene pair has guidance available from both CPIC and DPWG, the CPIC recommendations are detailed here. ^2^ = VKORC1 -1639G > A and CYP2C9 alleles are often combined together into an algorithm, alongside clinical variables, to guide initial warfarin dosing. Please note that CYP2C9 *1/*2 does not lead to recommended warfarin dose changes unless the VKORC1 -1639A allele is also present, and VKORC1 -1639GA does not lead to a dose change unless a CYP2C9 reduction-of-function allele is also present [9]. AD = alternative drug; CPIC = Clinical Pharmacogenetics Implementation Consortium; DPWG = Dutch Pharmacogenetics Working Group; LD = lower dose; ID = increase dose; M = consider additional monitoring (e.g., routine CK surveillance for simvastatin); PM = poor metaboliser; UM = ultra-rapid metaboliser; √ = guideline available; - = no guideline available.

**Table 2:** A non-exhaustive summary of recent interventional studies investigating pharmacogenomics of relevance to primary care. 1° = primary endpoint, 2° = secondary endpoints, RCT = randomised controlled trial, SoC = standard of care treatment.

Study	N	Genes	Treatment	Design	Duration	Intervention	Comparator	Endpoint	Outcome
Greden et al. 2019 (GUIDED) [12]	1167	Panel of 8 genes (including *CYP2C19* and *CYP2D6*)	SSRI, SNRI, TCA, other antidepressants, typical and atypical antipsychotics	RCT	24 weeks	PGx guided treatment	Standard of care treatment	1°–Symptoms (8 weeks) 2°–Response rate and remission (8 weeks)	1°–Symptom ↓ of 27.2% PGx vs. 24.4% SoC (*p* = 0.107) 2°–Response: 26.0% vs. 19.9% (*p* = 0.013) Remission: 15.3% vs. 10.1% (*p* = 0.007)
Perez et al. 2017 [13]	316	Panel of 30 genes (including *CYP2C19* and *CYP2D6*)	SSRI, SNRI, TCA, MAOI, other antidepressants	RCT	12 weeks	PGx guided treatment	Standard of care treatment	1°–% of patients with sustained response (12 weeks) 2°–Responder rate and side effect burden (12 weeks)	1°–38.5% PGx vs. 34.4% SoC (*p* = 0.4735) 2°–Responder rate: 47.8% vs. 36.1% (*p* = 0.0476) 2°–Side effect burden: 68.5% vs. 51.4% (*p* = 0.0260)
Bradley et al. 2018 [14]	685	Panel of 10 genes (including *CYP2C19* and *CYP2D6*)	SSRI, SNRI, TCA, other antidepressants, benzodiazepines, buspirone	RCT	12 weeks	PGx guided treatment	Standard of care treatment	Remission & response depression (12 weeks) Symptom severity & response anxiety (12 weeks)	Depression: Remission: 35% PGx vs. 13% SoC (*p* = 0.02) Response: 73% vs. 36% (*p* = 0.001) Anxiety: Symptom ↓ of 54% vs. 42% (*p* = 0.02) Response: 63% vs. 50% (*p* = 0.04)
Pirmohamed et al. 2013 (EU-PACT) [15]	455	*CYP2C9* and *VKORC1*	Warfarin	RCT	12 weeks	PGx guided treatment	Standard of care treatment	1°–% of time in INR range 2.0 to 3.0	1°–67.4% PGx vs. 60.3% SoC (*p* < 0.001)
Kimmel et al. 2013 (COAG) [16]	1015	*CYP2C9* and *VKORC1*	Warfarin	RCT	28 days	PGx guided treatment	Clinical dosing algorithm	1°–% of time in INR range 2.0 to 3.0	1°–45.2% PGx vs. 45.4% SoC (*p* = 0.91)
Gage et al., 2017 (GIFT) [17]	1650	*CYP2C9*, *VKORC1* and *CYP4F2*	Warfarin	RCT		PGx guided treatment	Clinical dosing algorithm	1°–composite of major bleeding, INR ≥ 4, death (all in 30 days) or VTE (in 60 days)	1°–10.8% PGx vs. 14.7% SoC (*p* = 0.02)
Pereira et al. 2020 (TAILOR-PCI) [18]	5302	*CYP2C19*	Clopidogrel	RCT	12 months	PGx guided oral P2Y12 inhibitor treatment	Standard of care (Clopidogrel)	1°–composite of cardiovascular death, myocardial infarction, stroke, stent thrombosis, and severe recurrent ischemia (12 months)	1°–in 4.0% PGx LOF carriers vs. 5.9% SoC LOF carriers (*p* = 0.06)
Claassens et al. 2019 (POPular) [19]	2488	*CYP2C19*	Clopidogrel	RCT	12 months	PGx guided oral P2Y12 inhibitor Tx	Standard of care (Ticagrelor or prasugrel)	1°–Net adverse clinical events (12 months) 1°–Major or minor bleeding (12 months)	Net events: 5.1% PGx vs. 5.9% SoC (*p* < 0.001 for noninferiority) Bleeding: 9.8% vs. 12.5% (*p* = 0.04)
Ko et al. 2015 [20]	2926	HLA-B*58:01	Allopurinol	Cohort	9 months	Allopurinol avoided in HLA-B*58:01 +ve patients	Allopurinol given in HLA-B*58:01-ve patients	1°–Incidence of SCARs in cohort compared to historical national average	1°–No cases of SCARs in prospective cohort (within 9 month follow up) vs. 7 cases to be expected based on historical average (0.3% per year), *p* = 0.0026
